# The effects of letrozole-induced maternal hyperandrogenism on sexual behaviors, testicular histology, and serum biochemical traits in male offspring rats: An experimental study

**DOI:** 10.18502/ijrm.v21i1.12669

**Published:** 2023-02-08

**Authors:** Zahra Shaaban, Amin Derakhshanfar, Mohammad Reza Jafarzadeh Shirazi, Mohammad Javad Zamiri, Javad Moayedi, Mahjoob Vahedi, Abouzar Valizadeh

**Affiliations:** ^1^Department of Animal Sciences, College of Agriculture, Shiraz University, Shiraz, Iran.; ^2^Diagnostic Laboratory Sciences and Technology Research Center, School of Paramedical Sciences, Shiraz University of Medical Sciences, Shiraz, Iran.; ^3^Center of Comparative and Experimental Medicine, Shiraz University of Medical Sciences, Shiraz, Iran.

**Keywords:** Androgens, Aromatase inhibitors, Rat, Sexual activities, Testes histopathology.

## Abstract

**Background:**

Intrauterine endocrine abnormalities have profound effects on the development of physiological disorders.

**Objective:**

This study aimed to assess the effects of in utero exposure to letrozole (an aromatase inhibitor) and its late consequences on the reproductive and metabolic performance of an adult male offspring.

**Materials and Methods:**

15 pregnant Sprague-Dawley rats (8 wk, 155 gr) were randomly assigned into 5 experimental groups (n = 3/each) and orally received either letrozole at doses of 0.25, 0.75, 1.00, and 1.25 mg/kg body weight (BW) or vehicle (control) on the gestation days of 16, 17, and 18. Pregnancy outcome, sexual behaviors on postnatal day 60, serum biochemical features, and the histopathology of testes were assessed in male offspring.

**Results:**

Compared to control group, delayed labor (21.83 vs. 24.25, p < 0.0001) and reduced litter size (n = 12.25 vs. n = 2, p < 0.0001) were recorded in 1.25 mg/kg BW group. A reduction in high-density lipoprotein level and the elevation of testes weight, BW gain, anogenital distance, as well as the serum concentrations of testosterone, triglycerides, cholesterol, and glucose were observed in 1.25 mg/kg BW (p < 0.0001) and 1.00 mg/kg BW (p < 0.0001) groups in comparison to control. A larger number of anogenital female sniffing, pursuit, and mounting behaviors were also observed in 1.25 mg/kg BW group in comparison to control (p < 0.0001). Severe testicular defects including necrosis and disruption of the epithelium of seminiferous tubules, sloughing of epithelial cells, and spermatogenesis arrest were observed in letrozole-treated groups, in a dose-dependent manner.

**Conclusion:**

Maternal exposure to letrozole can adversely affect the reproductive and metabolic performance of male offspring rats, suggesting an incomplete sex differentiation.

## 1. Introduction

Letrozole, a nonsteroidal aromatase inhibitor, has an antiestrogenic systemic effect due to the inhibition of estradiol production. It is a cheap drug with insignificant side effects and is used for ovulation induction in women with polycystic ovary syndrome (PCOS) and those undergoing in vitro fertilization/embryo transfer cycles (1). However, the efficacy and safety of letrozole are not clear (2). Pregnancy with ovulation induction drugs can create congenital anomalies in offspring that affect their health and function in adulthood. Letrozole treatment in infertile women created chromosomal anomalies in offspring (3). Although maternal hyperandrogenism in female animal models affected reproductive physiology and metabolic condition, data on male fetal programing by androgen excess was limited (4). In utero letrozole administration via the inhibiting aromatase activity can affect both the brain and reproductive organs (5), resulting in an impaired male sex differentiation during adulthood. Administration of letrozole on the gestational days (GDs) 21 and 22 in pregnant rat increased the testosterone levels in adulthood (75 days), and impaired sexual behaviors, especially ejaculation. In addition, prenatal letrozole treatment seemed to reduce libido among male rats, indicating the important role of aromatization in the development of normal sexual behavior in males (6). In another study, the prolonged use of letrozole delayed the seminiferous tubules' development (7).

Brain sexual differentiation depends on the level of testosterone or its metabolites at masculinization programming window (MPW), a critical period during fetal life (8). In rats, detectable levels of testicular testosterone occur on GD 15.5, with peak levels occurring on the GD 18.5 (9). Testicular testosterone surge on the GDs 16-18 is necessary for male rats' brain masculinization and defeminization (9, 10). Aromatization of fetal testosterone to estradiol is essential for the defeminization of the brain (11). Therefore, any defects in this mechanism during embryonic development may lead to impaired sexual differentiation, accompanied by the disruption of reproductive processes and normal male sexual behaviors in adults. (12). For instance, reduced anogenital distance (AGD) and testes size and development of reproductive disorders have been reported as a result of androgen abnormalities during the MPW (13). On the other hand, maternal hyperandrogenism is associated with rapid postnatal body weight (BW) gain, as observed in previous preclinical studies (14). Also, many chronic metabolic diseases in adulthood have been found to have an intrauterine or developmental origin (15).

To date, a comprehensive study has not been performed on the effects of maternal androgen on characteristics of metabolic syndrome and reproductive parameters in male rats. Therefore, the present study aimed to determine the effects of in utero exposure to letrozole and its consequences on testis histopathology, sexual behaviors, serum lipid parameters, and glucose concentration in male adult offspring.

## 2. Materials and Methods

### Animals 

In this experimental study, a total of 20 female Sprague-Dawley rats (130-180 gr, 8 wk) were obtained from the Center of Comparative and Experimental Medicine, Shiraz University of Medical Sciences, Shiraz, Iran. Female rats were maintained under standard conditions (12 hr light/dark cycle, temperature of 23 ± 3 C, and relative humidity of 25 ± 5%) with free access to rodent food and water. After adaptation to the new environment, 18 female rats in the proestrous or estrous phase were joined with sexually experienced adult male rats (mean weight: 375 gr) with a 2:1 female/male ratio overnight. In the morning after mating, a vaginal plug or spermatozoa in the vaginal smear was considered as the first day of pregnancy (GD1). 15 pregnant rats were randomly selected for letrozole administration during pregnancy.

### Experimental groups and treatments

Pregnant rats were housed in standard plastic cages individually. They were randomly divided into 4 experimental groups (n = 3/each) to orally receive letrozole at 4 doses (0.25, 0.75, 1.00, and 1.25 mg/kg BW) and a control group. Considering the reports on the embryotoxic effects of letrozole on pregnant rats and rabbits (16), a pilot study was performed to identify the doses above the physiological levels that were not lethal to the mother or the offspring. Accordingly, letrozole administration between 1.5 and 3.0 mg/kg BW caused fetal mortality, adsorption or death in early life, and uterine infection.

Letrozole (L6545, Sigma-Aldrich, St. Louis, USA) was dissolved in 1% carboxymethylcellulose (C5013, Sigma-Aldrich, St. Louis, USA) and was orally administered on days 16-18 of gestation (13). Testicular testosterone surge on 16-18 GDs is necessary for the brain masculinization and normal development of male rats (9, 10). The control rats were administrated with 1% carboxymethylcellulose on same days. The offspring's number, birth weight, and sex were recorded. The offspring remained with their mothers until weaning. At postnatal day (PND) 21, they were weaned, sexed, and weighed, and their AGD was measured. The anogenital distance index (AGDI) was calculated as AGD/BW × 100 (17). Male offspring (n = 4 per group) were kept in separate standard cages and were weighed weekly until the end of the study.

### Sexual behavior tests

Normal sexual behaviors, including mounting, erection, and intromission, were assessed on PND 60 after fertility evaluation. Sexually inexperienced male rats (8 male rats from the letrozole group (n = 4 per group) and 4 male rats from the control group) were investigated for natural copulatory behavior. The presence of the vaginal plug was regarded as an indicator of male rats' sexual experience (18). Sexual behavior tests were performed on male rats from 1.25 and 1.00 mg/kg BW letrozole and control groups. Male rats were individually housed in a cube-shaped glass cage (60 × 60 cm), and a non-receptive female was introduced after 10 min. In a dark room illuminated with red light, the investigatory (anogenital sniffing, sniffing, and pursuit) and sexual (mounting, intromission, and ejaculation) activities were recorded during a 30-min observation period (19).

### Tissue collection and blood sampling

Following the evaluation of sexual behaviors, male rats were euthanized (chloroform; Merck, KGaA, index No. 602-006-00-4, SigmaAldrich, USA), and blood samples were collected via cardiac puncture (blood volume: up to 5 ml) in a serum sep clot activator (item No. 476071, VACUETTEⓇ TUBE 5 ml, PREMIUM, Greiner Bio-One, Austria) without anticoagulants. Serum specimens were prepared after centrifugation at 3000 rpm for 15 min and were stored at -20 C until analysis for hormones and metabolites. Then, testes and epididymides were removed, weighed, and fixed in 10% buffer formalin for histological evaluation.

### Hormone and metabolite analyses

The serum testosterone concentration was measured by a competitive chemiluminescent enzyme immunoassay technique (IMMULITE 2000 System Analyzer, Catalog Number L2KTW2, Germany). In addition, the serum concentrations of glucose (Pars Azmun Kit Cat No. 98007, Iran), triglycerides (Biorexfars TRIGLYCERIDE, Product Code: BXC0271, Iran), low-density lipoprotein (LDL) (Biorexfars LDL DIRECT, Product Code: BXC0431, Iran), high-density lipoprotein (HDL) (Biorexfars HDL DIRECT, Product Code: BXC0421, Iran), and cholesterol (Biorexfars CHOLESTEROL, Product Code: BXC0261, Iran) were determined by colorimetric enzymatic procedures using ready-made reagents where the chemical compound quinoneimine was released during several chemical reactions. Quinoneimine can be measured photometrically and is directly related to the number of compounds under investigation.

### Histological evaluation of testes

Initially, a transverse section, which included the epididymis, was made through mid-testis. Then, 5-µm serial sections were dehydrated using graded concentrations of ethanol and xylene, embedded in paraffin, deparaffinized, and washed again with ethanol and xylene. The sections were then stained by a hematoxylin-eosin mixture. 3 sections were evaluated per animal under a light microscope (Motic Microscope, China) at 40 × , 100 × , 200 × , and 400 × magnification for objective analysis. Qualitative histological assessments were also performed to detect pathological conditions. The sections were then photographed by a digital camera (Nikon, NI_U 2013, Japan).

### Ethical considerations

Animal care and treatment were performed according to the guidelines of the local Ethics Committee of Shiraz University of Medical Sciences, Shiraz, Iran (Code: IR.SUMS.REC.1397.434).

### Statistical analysis

The SPSS 22 software for Windows (IBM SPSS Statistics for Windows, version 22, IBM Inc., Chicago, Illinois) was used to analyze the data. At first, the normality of the data was evaluated by Shapiro-Wilk and Kolmogorov-Smirnov normality tests. Mean separation was performed using Tukey's test following ANOVA (p < 0.05). Graphs were produced by GraphPad Prism, version 8.4.2 for Windows (GraphPad Inc., San Diego, CA, USA). The data have been presented as Mean ± Standard Error of Mean (SEM).

## 3. Results

### Pregnancy outcome

Compared to control group, the administration of letrozole at doses of 1.00 mg/kg BW and 1.25 mg/kg BW on GDs 16-18 increased the gestation length (p < 0.0001). Letrozole at a dose of 1.25 mg/kg BW led to a very small litter size in comparison to control group (p < 0.0001). However, letrozole treatment did not affect male/female ratio (p = 0.18) (Table I).

### Male offspring's BW, weaning weight, and testicular weight

The birth weight of male offspring was higher in the 1.25 mg/kg BW prenatal letrozole-treated group compared to the other groups (p = 0.04). The weaning weight at PND 21 was also significantly higher in the 1.25 and 1.00 mg/kg BW groups than the other experimental groups (0.75 and 0.25 mg/kg BW) (p < 0.0001). Moreover, the testicular weight was higher in 1.25, 1.00, and 0.75 mg/kg BW groups than 0.25 mg and control groups (p < 0.0001) (Table II).

### AGD, AGDI, and BW gain

AGD was significantly longer in the 1.25 and 1.00 mg/kg BW groups than in the other groups (p = 0.003). No significant differences were observed among the study groups regarding AGDI (p = 0.65) (Table II).

**Figure 1:**
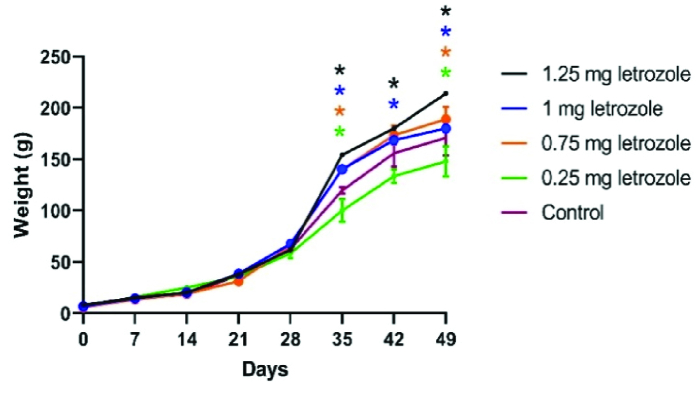
Body weight gain from birth to 7 wk of age in male offspring of the letrozole-treated pregnant rats (Mean ± SEM). Significant differences have been shown with stars. At each week, each group with a significant difference from the control group (p < 0.05) has been identified with its star.

The effects of time, treatment, and the interaction of time and treatment on BW gain were significant during the 7 wk of measurement (p < 0.0001). In the 6 ${}^{\text{th}}$ and 7 ${}^{\text{th}}$ wk, letrozole at doses of 1.25, 1.00, and 0.75 mg/kg BW significantly increased the BW gain compared to the control group and 0.25 mg/kg BW group (p = 0.002) (Figure 1).

### Sexual behavior

**Figure 2:**
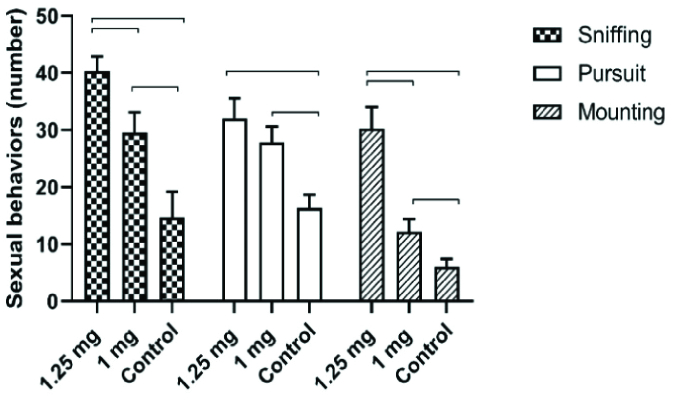
The number of anogenital sniffing, pursuit, and mounting behaviors in male offspring of the letrozole-treated pregnant rats (Mean ± SEM). The lines show significant differences between the groups at p < 0.05.

The frequency of female anogenital sniffing, pursuit and mounting behaviors was higher in the 1.25 mg/kg BW letrozole group than in the 1.00 mg/kg BW and control groups (p < 0.0001) (Figure 2).

### Serum testosterone, glucose, and lipid parameters 

The results indicated a dose-dependent increase in the serum concentration of testosterone, glucose, triglyceride and cholesterol. A remarkable increase was observed in the serum concentration of testosterone (p < 0.0001), glucose (p = 0.009), and triglyceride (p < 0.0001) in the 1.25 and 1.00 mg/kg BW letrozole groups compared to the other groups. Besides, male rats from the 1.25 mg/kg BW (p < 0.0001) and 1.00 mg/kg BW (p = 0.001) groups showed increased levels of cholesterol and decreased levels of HDL compared to the other groups. However, no significant difference was observed among the study groups regarding LDL levels (p = 0.7) (Table III).

### Histopathological findings

**Figure 3:**
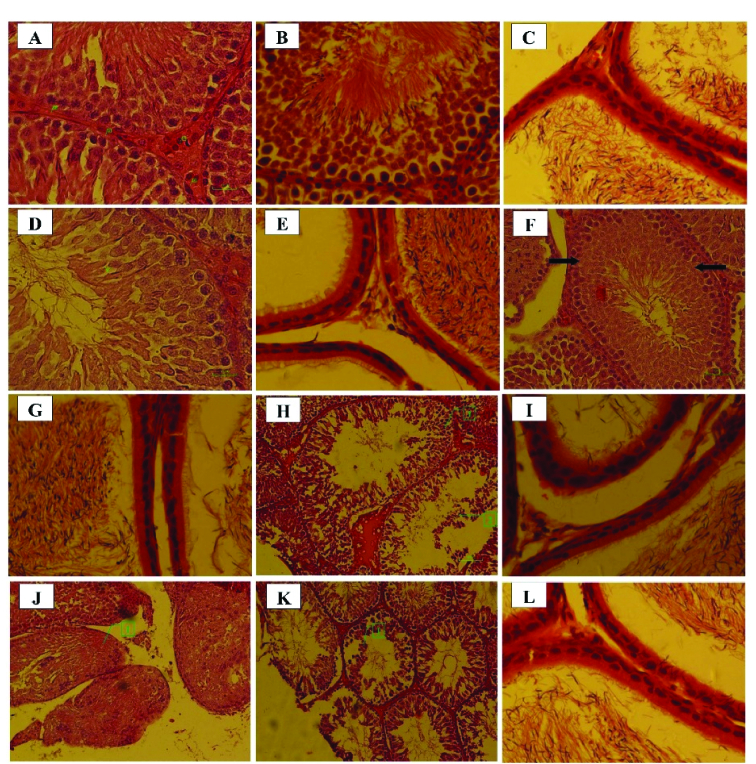
Histopathological findings in male offspring of the letrozole-treated pregnant rats. Control group: A) Normal structures of the interstitial cell (1), spermatogonium (2), basement membrane (3), and Sertoli cell (4) (H & E, × 400), B) Normal spermatogenesis (H & E, × 400), C) Normal structure of epididymis (H & E, × 400). Letrozole-treated group at a dose of 0.25 mg/kg BW: D) Necrotic spermatocytes with eosinophilic cytoplasm and karyolysis (arrow head) (H & E, × 400), E) Normal structure of epididymis (H & E, × 400). Letrozole-treated group at a dose of 0.75 mg/kg BW: F) Necrosis of the epithelium, spermatogenesis arrest, and karyolysis of the spermatocytes (arrow) (H & E, × 200), G) Normal structure of epididymis (H & E, × 400). Letrozole-treated group at a dose of 1.00 mg/kg BW: H) Severe necrosis and disruption of the epithelium of seminiferous tubules and severe spermatogenesis arrest (1), and moderate sloughing of epithelial cells due to necrosis of seminiferous tubules (2) (H & E, × 200), I) Normal structure of epididymis (H & E, × 400). Letrozole-treated group at a dose of 1.25 mg/kg BW: J) Severe necrosis and disruption of the epithelium of seminiferous tubules and severe spermatogenesis arrest (1) (H & E, × 100), K) Severe sloughing of epithelial cells due to necrosis of seminiferous tubules (1) (H & E, × 100), L) Normal structure of epididymis (H & E, × 400).

In the control group, interstitial cells, spermatogonium, basement membrane, and Sertoli cells had normal structures (Figure 3). Moreover, normal morphology of spermatogenesis with all generations of germ cells in the seminiferous epithelium was observed in the control group. Generally, the histopathological alterations of the testes in letrozole-treated groups were observed in a dose-dependent manner, being more severe in the 1.25 mg/kg BW group. Necrosis and disruption of the epithelium of seminiferous tubules were observed in all letrozole-treated groups; however, these changes were mild at a dose of 0.25 mg/kg BW, moderate at a dose of 0.75 mg/kg BW, and severe at doses of 1.00 and 1.25 mg/kg BW. Severe, moderate, and mild spermatogenesis arrest were also diagnosed in letrozole-treated groups at doses of 1.25, 1.00, and 0.75 mg/kg BW, respectively. Sloughing of epithelial cells due to necrosis of seminiferous tubules were observed in letrozole-treated groups at a dose of 1.25, 1.00, and 0.75 mg/kg BW in a dose-dependent manner. The normal structure of epididymis with high-sperm concentration was seen in all groups (Table IV).

**Table 1:** Gestation length, litter size, and sex ratio in letrozole-treated pregnant rats


	**Letrozole-treated (mg/kg BW)**			
**Parameters**	**Control**	**0.25**	**0.75**	**1.00**	**1.25**
		**Gestation length (days)**	21.0 ± 0.0 ${}^{\text{a}}$	21.0 ± 0.0 ${}^{\text{a}}$	22.0 ± 0.0 ${}^{\text{ab}}$	22.50 ± 0.29 ${}^{\text{b}}$	24.25 ± 0.14 ${}^{\text{c}}$
**Litter size (number)**	10.0 ± 0.0 ${}^{\text{ab}}$	9.50 ± 0.29 ${}^{\text{b}}$	10.0 ± 0.0 ${}^{\text{ab}}$	10.50 ± 0.29 ${}^{\text{a}}$	2.0 ± 0.0 ${}^{\text{c}}$
**Male/female ratio**	1.25 ± 0.14 ${}^{\text{a}}$	1.10 ± 0.07 ${}^{\text{a}}$	1.25 ± 0.14 ${}^{\text{a}}$	0.90 ± 0.04 ${}^{\text{a}}$	1.0 ± 0.0 ${}^{\text{a}}$
Data presented as Mean ± SD. Tukey's test following ANOVA. a, b, c: The means presented by similar letters are not significantly different (p > 0.05)					

**Table 2:** Birth weight, weaning weight, testes weight, anogenital distance and anogenital distance index in male offspring of the letrozole-treated pregnant rats


	**Letrozole-treated (mg/kg BW)**			
**Parameters**	**Control**	**0.25**	**0.75**	**1.00**	**1.25**
		**Birth weight (g)**	6.6 ± 0.3 ${}^{\text{a}}$	6.6 ± 0.3 ${}^{\text{a}}$	5.9 ± 0.03 ${}^{\text{a}}$	6.9 ± 0.05 ${}^{\text{a}}$	8.1 ± 0.1 ${}^{\text{b}}$
**Weaning weight (g)**	38.3 ± 0.6 ${}^{\text{a}}$	32.0 ± 0.5 ${}^{\text{b}}$	31.3 ± 0.8 ${}^{\text{b}}$	38.3 ± 1.2 ${}^{\text{a}}$	38.4 ± 0.2 ${}^{\text{a}}$
**Testes weight (g)**	1. 5 ± 0.02 ${}^{\text{a}}$	1.6 ± 0.08 ${}^{\text{a}}$	2.1 ± 0.008 ${}^{\text{b}}$	2.5 ± 0.07 ${}^{\text{c}}$	2.7 ± 0.005 ${}^{\text{c}}$
**AGD (mm)**	11.33 ± 0.3 ${}^{\text{ab}}$	9.8 ± 0.1 ${}^{\text{bc}}$	10.53 ± 0.2 ${}^{\text{ab}}$	12.23 ± 0.2 ${}^{\text{a}}$	11.8 ± 0.02 ${}^{\text{a}}$
**AGDI (mm/mg)**	38.3 ± 0.6 ${}^{\text{a}}$	30.7 ± 0.7 ${}^{\text{ab}}$	33.6 ± 0.2 ${}^{\text{b}}$	31.9 ± 0.7 ${}^{\text{ab}}$	30.9 ± 0.2 ${}^{\text{ab}}$
Data presented as Mean ± SE. Tukey's test following ANOVA. a, b, c: The means presented by similar letters are not significantly different by Tukey's test following ANOVA (p > 0.05). AGD: Anogenital distance, AGDI: Anogenital distance index					

**Table 3:** Serum levels of testosterone, glucose, triglycerides, cholesterol, HDL-cholesterol, and LDL-cholesterol in male offspring of the letrozole-treated pregnant rats


	**Letrozole-treated (mg/kg BW)**			
**Parameters**	**Control**	**0.25**	**0.75**	**1.00**	**1.25**
		**Testosterone (ng/ml)**	1.4 ± 0.1 ${}^{\text{a}}$	0.6 ± 0.08 ${}^{\text{b}}$	1.09 ± 0.06 ${}^{\text{ab}}$	7.5 ± 0.04 ${}^{\text{c}}$	10.22 ± 0.2 ${}^{\text{d}}$
**Glucose (mg/dl)**	119.3 ± 0.8 ${}^{\text{a}}$	128.0 ± 1.7 ${}^{\text{b}}$	125.7 ± 0.8 ${}^{\text{ab}}$	131.7 ± 0.8 ${}^{\text{b}}$	308.3 ± 0.6 ${}^{\text{c}}$
**Triglyceride (mg/dl)**	81.33 ± 0.6 ${}^{\text{a}}$	93.67 ± 2.1 ${}^{\text{b}}$	92.8 ± 2.0 ${}^{\text{b}}$	102.0 ± 4.04 ${}^{\text{c}}$	141.7 ± 0.6 ${}^{\text{d}}$
**Cholesterol (mg/dl)**	42.67 ± 0.3 ${}^{\text{a}}$	58.0 ± 2.6 ${}^{\text{b}}$	66.67 ± 0.8 ${}^{\text{c}}$	74.0 ± 2.3 ${}^{\text{c}}$	86.67 ± 0.8 ${}^{\text{d}}$
**HDL (mg/dl)**	25.67 ± 0.8 ${}^{\text{a}}$	25.0 ± 0.5 ${}^{\text{a}}$	24.67 ± 0.6 ${}^{\text{a}}$	19.0 ± 0.5 ${}^{\text{b}}$	13.67 ± 0.6 ${}^{\text{c}}$
**LDL (mg/dl)**	4.6 ± 0.3 ${}^{\text{a}}$	5.3 ± 0.8 ${}^{\text{a}}$	5.3 ± 0.3 ${}^{\text{a}}$	4.6 ± 0.8 ${}^{\text{a}}$	4.6 ± 0.6 ${}^{\text{a}}$
Data presented as Mean ± SE. Tukey's test following ANOVA. a, b, c, d: The means presented by similar letters are not significantly different (p > 0.05). HDL: High-density lipoprotein, LDL: Low-density lipoprotein					

**Table 4:** Histopathological findings in male offspring of the letrozole-treated pregnant rats


	**Letrozole-treated (mg/kg BW)**			
**Parameters**	**Control**	**0.25**	**0.75**	**1.00**	**1.25**
		**Interstitial cells (Leydig cells)**	N*	N	N	N	N
**Tubular basement membrane**	N	N	N	N	N
**Sertoli cells**	N	N	N	N	N
**Necrosis and disruption of the** **epithelium of seminiferous** **tubules**	N	+ ++	+++	+++
**Spermatogenesis arrest**	N	+ ++	+++	+++
**Sloughing of epithelial cells** **due to necrosis of** **seminiferous tubules**	- -	+ ++	+++
**Structure of epididymis**	N	N	N	N	N
N: Normal, +: Mild, ++: Moderate, +++: Severe					

## 4. Discussion

The present study findings indicated increased female anogenital sniffing, pursuit, and mounting behaviors in letrozole-treated groups. Exogenous testosterone treatment during the perinatal period adversely affected normal sexual behaviors (5). Prenatal letrozole administration at 56 µg/BW on GDs 10-22 led to homosexual partner preference in male rats (18). Furthermore, prenatal co-treatment of stress and letrozole during GDs 10-22 reduced the percentage of intromission and ejaculation in rats (20). In another study, daily treatment with 2.5 mg letrozole in adult rats for 2 wk alone or in combination with 1.00 g/kg testosterone every 2 wk reduced both investigatory and copulative behaviors. It resulted in a greater tendency toward exhibiting lordosis and female smelling (21). Male sexual behaviors include investigatory behaviors such as female anogenital sniffing. Pursuit is a social behavior before copulation that can occur toward a non-receptive female. Because no significant association exists among investigatory behaviors, the female pursuit may be a good marker for male mating readiness (17). Reduction in both investigatory and copulatory sexual behaviors was also reported in the rats treated with letrozole and testosterone plus letrozole (22). Overall, these findings suggest that maternal hyperandrogenism can interfere with the normal sexual behavior of male rats.

In the current study, a significant increase was detected in the serum level of testosterone in the offspring of the female rats prenatally receiving 1.25 and 1.00 mg/kg BW letrozole. Males and females exposed to androgen excess during intrauterine life showed an increment in circulating testosterone levels during adulthood (6, 23, 24). Letrozole-treated adult mice showed elevated levels of serum testosterone (25). Furthermore, letrozole treatment at 2.5 mg daily for 4 months resulted in a noticeable increase in the testosterone level and the testosterone/estrogen ratio amongst males with idiopathic infertility (26). Increased testosterone levels seem to be an inevitable effect of androgenic treatments, which is not affected by the timing of androgenic treatment. Moreover, AGD as an indicator of androgen action during the prenatal period is greatly affected by changes in the androgen level or action, especially during intrauterine masculinization (17). The findings of the present study revealed an increase in the AGD of letrozole-treated groups compared to the control group, which represented the direct effects of letrozole.

In the current study, severe testicular injuries were observed in 1.25 and 1.00 mg/kg BW groups. The undesirable changes in testicular morphology were necrotic seminiferous tubules, disruption of the epithelium of seminiferous tubules, and sloughing of epithelial cells due to necrosis of seminiferous tubules. The reproductive system is extremely sensitive to exogenous manipulations, particularly abnormalities in sex steroids during the MPW (4). Increased androgen exposure during fetal sex differentiation affected seminiferous tubule structure, and germ cell count in rams (27), rats (4), and Galea spixii (7). Previous findings demonstrated that chronically letrozole-treated adult male rats showed several defects, such as sloughed germ cells, late pachytene spermatocyte, spermatid in the lumen, empty area due to sloughed germ cells, invagination of lamina propria in some seminiferous tubules, and vacuolization of intraepithelial cells in seminiferous tubules (4, 27, 28). Although the exact mechanism of these testicular pathologies was not reported, the existence of the empty area resulted in intraepithelial vacuolization and denuded epithelium. Moreover, estrogen deficiency induced by letrozole might disrupt the adhesions between the germ and Sertoli cells, resulting in the loss of premature germ cells and the necrotic structure of seminiferous tubules and finally spermatogenesis arrest (4, 27, 28). Impaired spermatogenesis in adulthood was found in anastrozole-treated male rats (22). Similarly, knockout mice for *cyp19* gene (ArKO), although initially fertile, developed spermatogenesis arrest at 1 yr old (29). The obvious morphological feature of the spermatogenesis arrest in the early stages is an increase in apoptosis, a reduction in the round and elongated spermatids, without any changes in the Sertoli cells and earlier germ cells (29). Spermatogenesis arrest, as a post-pubertal change in testes, demonstrated the crucial role of estrogen/androgen balance in maintaining normal testicular morphology and spermatogenesis (30). Our findings also showed the spermatogenesis arrest as characterized by necrosis and disruption of the epithelium of seminiferous tubules in prenatally letrozole-treated male rats that highlighting the critical role of estrogen in normal male reproduction.

In the current research, male offspring of the female rats treated with 1.25 mg/kg BW letrozole were heavier at birth. Furthermore, higher testicular weight and increased BW gain were recorded in male offspring of the 1.25 and 1.00 mg/kg BW groups. The weight gain from birth to early childhood can be associated with androgen excess during intrauterine life (14). Previous studies showed that chronic exposure to aromatase inhibitors led to defects in testicular weight, serum testosterone levels, and testicular structure in rats (22, 31). Moreover, long-term administration of aromatase inhibitors in the postnatal period can increase weight gain, which could be true for prescribing letrozole before birth, as seen in the present research.

Transient prenatal androgen administration on GDs 16-19 led to increased BW, serum triglycerides and cholesterol levels in adult female rats, which are observed in our study (32). In the present research, increased serum cholesterol and triglycerides and decreased HDL levels were observed due to 1.25 and 1.00 mg/kg BW letrozole administration. Furthermore, in the current study, increased androgen levels due to aromatase inhibition by letrozole created testosterone-like effects on increased serum glucose levels. Prenatal exposure to testosterone caused elevated serum glucose levels in contrast to prenatal dihydrotestosterone (non-aromatizable androgen) treatment in male rats. Aromatization and conversion of testosterone to estradiol may alleviate this negative effect of glucose elevation (33). The alteration in glucose homeostasis directly results from prenatal androgen. The adverse effects of androgen excess during the critical period of intrauterine life on metabolic traits were partially evident.

The present study findings showed that exposing pregnant rats to letrozole delayed the delivery and decreased the litter size at dose 1.25 mg/kg BW. Generally, 2 hypotheses have been proposed to explain the effect of letrozole on the gestation length and litter size: 1) direct action of androgens and 2) letrozole-induced estrogen reduction. Estrogen levels were found to increase in rats during GDs 14-21 (34) and labor (35). Consistently, late delivery was found to occur due to the subcutaneous administration of testosterone propionate at doses greater than 1.00 mg/kg BW on GDs 14-19 (36) and due to prenatal letrozole treatment on GDs 15-21. Letrozole treatment at 0.02 mg/kg on GDs 15-21 also reduced the litter size in rats (37). Therefore, lower estrogen levels induced by letrozole can be detrimental to fetal viability and pregnancy outcomes, including the gestation length, as proven in the previous studies.

## 5. Conclusion

In summary, letrozole treatment of the female rats during pregnancy exerted undesirable peripheral and reproductive effects on their male offspring. In this male rat model of hyperandrogenism, 1.25 and 1.00 mg/kg BW doses of letrozole administrated on GDs 16-18 exerted strong androgenic effects on male fetuses, including complete necrosis of seminiferous tubules, disrupted normal sexual behaviors, and increased serum testosterone, cholesterol, triglycerides, and glucose levels. The results suggested the noticeable potential of letrozole to cause fundamental changes in the prenatal hyperandrogenic rat model.

##  Conflict of Interest

The authors declare that the research has been conducted without any commercial or financial relationships that could be construed as a potential conflict of interest.

## References

[B1] Olulana DI, Popoola C (2021). Comparative effects of pre-gestational doses of clomiphene citrate versus letrozole on the heart of developing wistar rats. Anat J Afr.

[B2] Kar S (2013). Current evidence supporting “letrozole” for ovulation induction. J Hum Reprod Sci.

[B3] Yun J, Choi YS, Lee I, Won YB, Lee JH, Seo SK, et al (2018). Comparison of congenital malformations among babies born after administration of letrozole or clomiphene citrate for infertility treatment in a Korean cohort. Reprod Toxicol.

[B4] Ramezani Tehrani F, Noroozzadeh M, Zahediasl S, Ghasemi A, Piryaei A, Azizi F (2013). Prenatal testosterone exposure worsen the reproductive performance of male rat at adulthood. PloS One.

[B5] Martin YH, Sc Thesis].

[B6] Gerardin DCC, Piffer RC, Garcia PC, Moreira EG, Pereira OCM (2008). Effects of maternal exposure to an aromatase inhibitor on sexual behaviour and neurochemical and endocrine aspects of adult male rat. Reprod Fertil Dev.

[B7] Arroyo MAM, Silva Santos PRD, de Oliveira MF, de Assis AC (2021). Prolonged use of letrozole causes morphological changes on gonads in Galea spixii. Anim Reprod.

[B8] Onaolapo AY, Onaolapo OJ (2022). A Review of the Impact of Testosterone on Brain and Aging-related Decline in Brain Behavioural Function. Frontiers in Clinical Drug Research-CNS and Neurological Disorders.

[B9] Macleod DJ, Sharpe RM, Welsh M, Fisken M, Scott HM, Hutchison GR, et al (2010). Androgen action in the masculinization programming window and development of male reproductive organs. Int J Androl.

[B10] Reznikov A, Nosenko N, Tarasenko L (2004). Early postnatal effects of prenatal exposure to glucocorticoids on testosterone metabolism and biogenic monoamines in discrete neuroendocrine regions of the rat brain. Comparative Biochemistry and Physiology Part C: Toxicology & Pharmacology.

[B11] Cara AL, Henson EL, Beekly BG, Elias CF (2021). Distribution of androgen receptor mRNA in the prepubertal male and female mouse brain. J Neuroendocrinol.

[B12] Hughes IA, Deeb A (2006). Androgen resistance. Best Practice & Research Clinical Endocrinology & Metabolism.

[B13] Dean A, Smith LB, Macpherson S, Sharpe RM (2012). The effect of dihydrotestosterone exposure during or prior to the masculinization programming window on reproductive development in male and female rats. Int J Androl.

[B14] Huang G, Aroner SA, Bay CP, Gilman SE, Ghassabian A, Loucks EB, et al (2021). Sex-dependent associations of maternal androgen levels with offspring BMI and weight trajectory from birth to early childhood. J Endocrinol Invest.

[B15] Barker DJP (2007). The origins of the developmental origins theory. Journal of Internal Medicine.

[B16] Tiboni GM, Marotta F, Rossi C, Giampietro F (2008). Effects of the aromatase inhibitor letrozole on in utero development in rats. Hum Reprod.

[B17] Shaaban Z, Tamadon A, Jafarzadeh Shirazi MR, Zamiri MJ, Derakhshanfar A (2022). Maternal aromatase inhibition via letrozole altered RFamide-related peptide-3 and gonadotropin-releasing hormone expression in pubertal female rats. Iran J Basic Med Sci.

[B18] Olvera-Hernández S, Chavira R, Fernández-Guasti A (2015). Prenatal letrozole produces a subpopulation of male rats with same-sex preference and arousal as well as female sexual behavior. Physiol Behav.

[B19] Chu X, Ågmo A

[B20] Hernández A, Olvera-Hernández S, Fernández-Guasti A (2020). Lack of interaction between prenatal stress and prenatal letrozole to induce same-sex preference in male rats. Physiol Behav.

[B21] Vari CE, Ősz BE, Perian M, Mărușter MS, Miklos A, Bosa P, et al

[B22] Turner KJ, Morley M, Atanassova N, Swanston ID, Sharpe RM (2000). Effect of chronic administration of an aromatase inhibitor to adult male rats on pituitary and testicular function and fertility. J Endocrinol.

[B23] Chinnathambi V, Balakrishnan M, Yallampalli C, Sathishkumar K (2012). Prenatal testosterone exposure leads to hypertension that is gonadal hormone-dependent in adult rat male and female offspring. Biol Reprod.

[B24] Abdelaziz AS, Kamel MA, Ahmed AI, Shalaby SI, El-Darier SM, Beshbishy AM, et al (2020). Chemotherapeutic potential of Epimedium brevicornum extract: The cGMP-specific PDE5 inhibitor as anti-infertility agent following long-term administration of tramadol in male rats. Antibiotics.

[B25] Verma R, Krishna A (2017). Effect of Letrozole, a selective aromatase inhibitor, on testicular activities in adult mice: Both in vivo and in vitro study. Gen Comp Endocrinol.

[B26] Peivandi S, Jafarpour H, Abbaspour M, Ebadi A (2019). Effect of letrozole on spermogram parameters and hormonal profile in infertile men: A clinical trial study. Endocr Regul.

[B27] Rojas-García PP, Recabarren MP, Sarabia L, Schön J, Gabler C, Einspanier R, et al

[B28] Bormann CL, Smith GD, Padmanabhan V, Lee TM (2011). Prenatal testosterone and dihydrotestosterone exposure disrupts ovine testes development. Reproduction.

[B29] Robertson KM, O’Donnell L, Jones ME, Meachem SJ, Boon WC, Fisher CR, et al (1999). Impairment of spermatogenesis in mice lacking a functional aromatase (cyp 19) gene. Proc Natl Acad Sci USA.

[B30] Misiakiewicz K, Kolasa A, Kondarewicz A, Marchlewicz M, Wiszniewska B (2013). Expression of the c-Kit receptor in germ cells of the seminiferous epithelium in rats with hormonal imbalance. Reprod Biol.

[B31] Kondarewicz A, Kolasa A, Zawiślak B, Baranowska-Bosiacka I, Marchlewicz M, Wenda-Różewicka L, et al (2011). Testis morphology in rats chronically treated with letrozole, an aromatase inhibitor. Folia Histochem Cytobiol.

[B32] Demissie M, Lazic M, Foecking EM, Aird F, Dunaif A, Levine JE

[B33] Lazic M, Aird F, Levine JE, Dunaif A (2011). Prenatal androgen treatment alters body composition and glucose homeostasis in male rats. J Endocrinol.

[B34] Agoreyo FO, Okeke OG (2014). Quantitative evaluation of serum oestrogen levels in the three trimesters of pregnancy in albino rat. NISEB Journal.

[B35] Tiboni GM, Ponzano A (2016). Fetal safety profile of aromatase inhibitors: Animal data. Reprod Toxicol.

[B36] Wolf CJ, Hotchkiss A, Ostby JS, LeBlanc GA, Gray Jr LE (2002). Effects of prenatal testosterone propionate on the sexual development of male and female rats: A dose-response study. Toxicol Sci.

[B37] Kafali H, Iriadam M (2007). A novel tocolytic agent: Effects of letrozole on gestational length and parturition time. Am J Perinatol.

